# Interior frontiers and highly skilled migrants' work-related challenges in Japan

**DOI:** 10.12688/f1000research.147767.1

**Published:** 2024-04-29

**Authors:** Liang Morita

**Affiliations:** 1 Nagoya University, Nagoya, Japan

**Keywords:** Japan, immigration, highly skilled migrants, workplace, interior frontiers

## Abstract

In this opinion article, the author argues that highly skilled migrants in Japan face many challenges and are ineffectively used due to the existence of interior frontiers. Although interior frontiers are more subtle than the external boundaries these migrants have had to cross to enter the country, they have tremendous power over their everyday lives. Ethnocentric attitudes, influenced by *Nihonjinron*, have contributed to the existence of these frontiers. They emphasise homogeneity instead of flexibility and accommodation, and exist in the workplace. Employers want a homogeneous workplace and highly skilled migrants face strong pressures to assimilate. There is also an insistence on Japanese human resource practices such as seniority-based pay and promotion. An interior frontier also exists in the hiring process, and migrants are less likely to progress beyond the first round of interviews. With Japan’s rapidly aging and declining population and acute labour shortages, there is urgency in addressing these frontiers.

## 1. Introduction

Since the end of the 20 ^th^ century, many developed countries have focused on having knowledge-based economies, prompting them to participate in the race to attract highly skilled migrants ( Shachar, 2006). Japan has also joined this global competition for highly skilled migrants, and frames these migrants as desirable human resources ( Joppke, 2021).

Japan has been selectively prioritising the highly skilled from the late 1980s ( Liu-Farrer et al., 2023). In 2012, it introduced a point-based system and the category of ‘highly skilled professionals’, providing incentives for the highly skilled to live and work in the country. The government has developed increasingly attractive policies and programmes to recruit and retain these migrants, making Japan the most liberal immigration regime ( Oishi, 2014). From the beginning of the 21 ^st^ century, Japanese companies and recruitment agencies have tapped into foreign labour markets, setting up job fairs and collaborating with universities in Southeast Asia to recruit their graduates ( Conrad and Meyer-Ohle, 2019).

In spite of lawmakers’ and policymakers’ good intentions, highly skilled migrants do not necessarily find Japan to be an attractive destination and those who have come do not necessarily integrate smoothly into the workplace or are able to find alternative employment when they wish to. In fact, research has shown that the country can be unattractive ( Morita, 2017; Liu-Farrer, 2023), highly skilled migrants face challenges in the workplace ( Hof and Tseng, 2021), and there is discrimination in the hiring process ( Igarashi and Mugiyama, 2023). In the context of Japan’s rapidly aging and declining population and shrinking workforce, there is urgency in understanding and addressing the issues involved.

This opinion article draws on the concept of interior frontiers in Lems’ (2020a, 2020b) work on young asylum seekers in Switzerland. In spite of the fact that these young people are already physically present in the country, interior frontiers prevent them from being accepted and integrated into Swiss society. Likewise, highly skilled migrants in Japan face interior frontiers in the workplace, although they have successfully crossed the external boundary and entered the country. There are parallels between Lems’ work and the situation of these migrants in Japan that can help us better understand and address the challenges the latter face.

The next section introduces the concept of interior frontiers and ethnocentric attitudes, followed by employers’ desire for a homogeneous workplace in Section 3. Highly skilled migrants face strong pressures to assimilate into the workplace, and their unique skills and abilities are undervalued. Section 4 discusses employers’ insistence on Japanese human resource practices such as seniority-based pay and promotion. Section 5 finds discrimination in the hiring process, and the paper ends with Discussion and concluding remarks in Section 6.

## 2. Interior frontiers and the ethnocentric attitudes that contribute to their existence

### 2.1 Interior frontiers

‘Interior frontier’ (also ‘interior border’ or ‘interior boundary’) was originally a concept put forward by Johann Fichte, a German philosopher in the 19 ^th^ century. It was revived by Etienne Balibar about 30 years ago, and was employed by Stoler (2017, 2018) to describe the rise of Trumpism in the US when Donald Trump was President from 2017-2021. According to Fichte, an interior frontier is a fortifying moral barricade against the erosion of the nation and self, a boundary between those who belong in the nation and those who do not ( Stoler, 2017). Stoler stresses that these barricades and boundaries result in vast inequalities ( Morita, 2023).

Lems (2020a, 2020b), in adopting the concept in her work on young asylum seekers from Eritrea, Guinea and Somalia in Switzerland, describes interior frontiers as vernacular thresholds of belonging that create unspoken distinctions between self and other, familiar and alien, and inside and outside. They are not conspicuous frontiers created by overt acts of boundary-drawing, but are put in place in much more subtle ways. In spite of the fact that interior frontiers are less visible than external ones, they have tremendous power over the life-worlds of individuals, as they determine who is allowed in and who is kept out of society ( Lems, 2020a, 2020b; Morita, 2023).

There are striking parallels between the following account provided by Lems and what highly skilled migrants in Japan face at the workplace. In the school which young asylum seekers attend, these young people are taught to integrate into a system of values which is extremely alien to them. The approach used by their teachers in this process of integration is one-way, which means that the young people are required to fully submerge into Swiss society, but the Swiss do not have to show any flexibility in accommodating them. In this asymmetrical power relationship, even if the young people were to obey the rules and try to blend in, acceptance as members of Swiss society is not guaranteed ( Lems, 2020a, 2020b; Morita, 2023).

The young asylum seekers studied by Lems have in fact experienced rejection in spite of their efforts in submitting to the rules and blending in. They frequently experience being excluded by the Swiss, they have difficulties in transferring from their school’s ‘integration’ stream to ‘regular’ stream, and they have trouble securing apprenticeships ( Lems, 2020a, 2020b).

### 2.2 Ethnocentric attitudes that contribute to the existence of interior frontiers

In the author’s opinion, ethnocentric attitudes have contributed to the existence of interior frontiers, and these attitudes have been strongly influenced by *Nihonjinron* (‘theories about being Japanese’). *Nihonjinron* has been referred to as Japan’s dominant identity discourse ( Befu, 2001), and has been the subject of thousands of books and articles ( Rear, 2017). Its key tenets, summarised by Rear (2017), are firstly, that Japan is a homogeneous country and its culture and people are extremely unique, to the point of its culture being superior to that of other countries. Secondly, Japan is a vertical society in which social obligations, indebtedness and shame are prioritised over Western values of individual rights, duties and conscience. Thirdly and finally, Japanese culture values harmony over conflict and emotion over rationality.

Homogeneity is emphasised, along with the unique set of collectivist and harmonious social values that have built the country ( Rear, 2017). Japanese values such as self-sacrifice and supposed to be truly understood only by the Japanese, because they possess a unique sensibility. If one deviates from Japanese values, for example, by behaving in a manner deemed to be individualistic or self-serving, one risks being judged as having betrayed what it truly means to be Japanese ( Rear, 2017).

Ethnocentric attitudes influenced by *Nihonjinron* are unfriendly to migrants in general, to say the least. The emphasis on homogeneity translates into the thinking that Japan is for the Japanese only, and that non-Japanese do not belong. The belief that Japanese culture and customs are unique and superior encourages an insistence on doing things the Japanese way instead of openness, flexibility and accommodation in situations where non-Japanese are involved ( Morita, 2022). We will see what this looks like in real life in the workplace when employers insist on Japanese practices and are unwilling or unable to accommodate highly skilled migrants.

## 3. Employers want a homogeneous workplace

We begin this section with the experiences of fresh foreign university graduates who joined Japanese companies at entry level. We will see that they face pressures to assimilate culturally. A boundary between the Japanese and non-Japanese exists, although more subtle and less overt and conspicuous than the boundaries the latter have had to cross to enter the country and later the company. This is an interior frontier which has great power over the life-worlds of individuals ( Lems, 2020a, 2020b), as we will see below.

In the application process, fresh foreign university graduates encounter extremely high ethnocentric cultural expectations ( Liu-Farrer and Shire, 2021). In spite of the discourse of workplace diversity resulting in higher revenues and greater success, most companies expect highly skilled migrants to conform culturally and behave like Japanese employees, referred to as ‘coerced harmonisation’ by Wakisaka (2018). Companies recruit these migrants in order to assimilate them into Japanese employees ( Hof and Tseng, 2021). In this process of assimilation, non-Japanese are measured against the ideal Japanese employee, assuming that non-Japanese are imperfect versions of the Japanese ( Desai-Trilokekar et al., 2016) rather than ‘global talent’ they are often said to be. Some white employees feel that they are not adequately valued for their skills and abilities, and have been hired merely for their physical appearances, which serve as symbols of diversity ( Hof, 2021). All of the above: pressures to conform culturally and assimilate, being thought of as of less worth than Japanese coworkers, and having one’s skills and abilities undervalued, directly impact highly skilled migrants’ everyday lives. It is widely known in business management research that employees have a need to feel unique, to maintain a distinctive and differentiated sense of self ( Shore et al., 2011). For many highly skilled migrants, this need is probably not being met.

What comes across clearly in the preceding paragraph is employers’ desire for a homogeneous workplace, which is directly related to the *Nihonjonron* emphasis on a homogeneous Japan. This desire is carefully detailed in Hof and Tseng’s (2021) study, which found a homogenising work culture, a sole focus on assimilation, and inflexibility towards others. Homogeneity in fact supersedes the desire for a more diverse workplace, where employees from different backgrounds can better serve customers around the world in this age of globalisation. This can be seen in the tendency of companies to hire highly skilled migrants whom they think can be ‘Japanised’ or made to behave like the Japanese ( Wakisaka, 2018). This is in fact a more important criterion in the hiring process than the unique skills and abilities an applicant possesses.

Cox (1991) and Joshi’s (2006) model in business management can help us better understand where Japanese companies stand now and where they should be ideally. The model is made up of demographically heterogeneous organisations in three stages, each stage being different in terms of the proportion of diverse social groups, their integration, and power and status hierarchy. The stages are named ‘low’ (monolithic), ‘moderate’ (pluralistic) and ‘high’ (multicultural). ‘Monolithic’ has the lowest degree of heterogeneity and structural integration, while ‘multicultural’ has the highest.

Most Japanese companies fall into the category of ‘monolithic’ ( Sekiguchi et al., 2016; Ghosh et al., 2023). There are distinct in-groups and out-groups in which in-group members (Japanese) are treated preferentially while out-group members (non-Japanese) are disregarded. In monolithic organisations, employees from dominant groups possess high status and power, hold positions at higher levels, and command more respect and deference ( Ghosh et al., 2023). From the point of view of non-dominant employees, the impermeability of status and power differences threatens their identity and devalues their sense of self, and their uniqueness needs are not met in this exclusionary environment. More importantly, non-dominant employees’ unique perspectives, knowledge or ideas are not considered as relevant ( Shore et al., 2011). They are not treated as insiders who belong and whose uniqueness is valued.

At the other end of Cox (1991) and Joshi’s (2006) model is ‘multicultural’ organisations. These companies are heterogeneous, structurally integrated, and inclusive. All employees respect, value, and learn from one another ( Ghosh et al., 2023). Employees from all social groups are treated as insiders and encouraged to retain and leverage their unique attributes. Non-dominant employees can effectively use their unique knowledge, skills and abilities, and are assured of being valued members of the organisation and that it is safe to express their identities and associated values ( Ghosh et al., 2023).

The description in the preceding paragraph contrasts sharply with the situation of highly skilled migrants in Japanese companies, where they are expected to conform culturally and behave like Japanese employees. They are also measured against Japanese employees and made to feel second-rate, and their skills and abilities undervalued. Japanese companies would be far more productive if they are willing to learn from Cox (1991) and Joshi’s (2006) ‘multicultural’ organisations.

## 4. Employers insist on Japanese human resource practices

Many of the frequently discussed workplace practices that highly skilled migrants are dissatisfied with stem from the tradition of lifelong employment in Japan ( Liu-Farrer, 2023). While it is understandable that Japan has developed its own system of human resource practices, what comes across clearly is employers’ unwillingness or inability to be flexible and accommodate their non-Japanese employees ( Liu-Farrer, 2023). This suggests the existence of an interior frontier, where one-way submission to the rules is expected of outsiders, while insiders do not have to show any flexibility in accommodating them ( Lems, 2020a; Lems, 2020b).

Japanese companies mostly recruit fresh university graduates with a view to employing them for the rest of their working lives. Employees who join companies mid-career are relatively few, and often find that they lack the in-depth knowledge of the company possessed by coworkers who have been working there since the beginning of their careers. Many highly skilled migrants are mid-career when hired by Japanese companies, and feel left out of the unwritten rules and conventions shared by the majority of their Japanese colleagues ( Morita, 2022).

In comparison with other developed countries, Japanese salaries may appear low, due to the fact that Japanese salaries are determined with the assumption that employees stay in the same company for their entire working lives. Salaries start low but gradually increase with annual increments. Highly skilled migrants are often dissatisfied with their earnings and this is exacerbated by their tendency to leave before they benefit from the yearly increments ( Morita, 2022).

Promotion is seniority-based, depending on the length of time employees have served the company, which contrasts sharply with performance-based promotion practised in many developed countries. Many highly skilled migrants feel that promotion is slow ( Liu-Farrer and Shire, 2021), and their career development stifled.

Company-provided training is yet another practice which highly skilled migrants are dissatisfied with. This directly impacts fresh foreign university graduates who join Japanese companies alongside their Japanese counterparts. Companies typically provide three to six months’ training for their new employees, the contents of which are company-specific and not transferrable or useful should the employee choose to work for a different employer. From the point of view of highly skilled migrants, the skills gained are too specific and may hinder their mid-career moves to other companies or other countries, thereby limiting them to their initial places of employment.

In general, Western-style human resources practices such as performance-based pay and promotion have not become mainstream among Japanese companies ( Haghirian, 2022). In addition to benefitting highly skilled migrants, it is in these companies’ best interests to modernise their practices, as explained below.

Due to Japan’s rapidly aging and declining population, there has been an acute shortage of skilled labour ( Haghirian, 2022). Skilled employees are in short supply in many new business areas (including technology-related ones, digitisation and social media), and the conventional practice of giving employees a few years to develop the necessary skills and knowledge has proven to be too slow. Employers realise that they need skilled employees in fast-moving business fields, but find it very difficult to recruit mid-career employees in the Japanese labour market. The difficulties are due to relatively low numbers of Japanese employees changing jobs mid-career, and to the traditional seniority system being an obstacle to the integration of new employees of higher rank. Experienced and skilled employees are reluctant to change employers in case they end up in a lower position than they should in the seniority hierarchy of the new company ( Haghirian, 2022).

In this context of a fast-moving and everchanging business world, it would make business sense, or would even be imperative to overhaul Japanese human resource practices. The traditional training system for new employees and seniority-based pay and promotion have to adapt to changing times and circumstances.

## 5. Discrimination in the hiring process

Compared with studies conducted on highly skilled migrants and the challenges they encounter at the workplace, there are relatively few on discrimination in the hiring process. Igarashi and Mugiyama, 2023 is one of the most recent, which includes migrants of all skill levels and utilises the concept of taste-based discrimination. The authors found that there is taste-based discrimination in the hiring process, which suggests the existence of an interior frontier when migrants seek employment.

Taste-based discrimination is defined as personal and societal (i.e. customers and coworkers) preferences for native applicants, and is due to prejudice against other ethnic groups ( Igarashi and Mugiyama, 2023). Becker (1957), who first put forward this notion, stated that employers avoided hiring from other ethnic groups in order to avoid interacting with them, and they assumed that customers and coworkers are prejudiced against these groups. By not having employees from other ethnic groups, employers felt they were protecting their companies’ reputation and avoiding conflict between their employees and those from other ethnic groups that may decrease workplace productivity ( Igarashi and Mugiyama, 2023).

Igarashi and Mugiyama found that due to taste-based discrimination, non-Japanese applicants are less likely to progress to the next stage of the job application process (after the initial round of interviews) after controlling for human capital.

‘Prejudice’ is a key word in Igarashi and Mugiyama’s work, which is generally understood as an unfair or unreasonable opinion or feeling against someone or something. It is difficult to say with certainty as the authors did not elaborate on prejudices of Japan employers, but prejudice against other ethnic groups would point to a boundary between those who belong in the nation and those who do not ( Stoler, 2017), or between insiders and outsiders ( Lems, 2020a; Lems, 2020b). Ethnocentric attitudes influenced by *Nihonjinron* also appear to be involved, since employers clearly prefer a homogeneous workplace without employees from other ethnic groups.

The preference for a homogeneous workplace is also clear in a government publication on Japan’s labour situation ( JILPT, 2014). About two-thirds of employers who participated in a survey said they had no plans to hire foreign employees. This is a telling response in the context of Japan’s rapidly aging and declining population and labour shortages.

In the same publication, it is reported that in companies which are experienced in hiring foreign employees, Japanese language ability is prioritised over specialist knowledge, skills or experience ( JILPT, 2014). This is justified by the explanation that work-related instructions are given in Japanese, so Japanese language skills are a precondition for employment.

The Japanese language requirement sounds commonsensical and reasonable, but it is also a fact in Japan that practically all students study English in elementary, middle and high schools. Those who attend university receive a few more years of English language instruction. With English being the global lingua franca, and provided Japanese employers are willing to make the effort, communication with non-Japanese employees should be possible to some extent. By laying down the rule that Japanese language is a precondition for employment, employers are revealing their expectations of migrants’ one-way submission to Japanese rules.

## 6. Discussion and concluding remarks

The Japanese situation involving highly skilled migrants in the workplace is not unique in the world. In the literature on diversity management, lack of preparedness on the part of human resource staff members to accommodate and make efficient use of highly skilled migrants’ diversity is nothing new ( van Riemsdijk and Basford, 2022). Friesen and Ingram (2013) noted managers’ lack of diversity management experience in Canadian engineering firms. Managers with limited intercultural management experience complained about the ‘deficits’ of foreign engineers vis-a-vis their Canadian counterparts initially, but in the long run, they learned that foreign engineers’ differences (not deficits) can be a strength.

Diversity of experience can increase productivity and innovation for companies, and imparts new skills that enhance professional development for employees ( van Riemsdijk and Basford, 2022). Openness to diversity is crucial to cross-cultural collaboration in the workplace, which requires employer commitment to diversity management ( Homan et al., 2008).

In a study conducted in Sweden ( Morillas and Romani, 2023), managers who have been encouraged to believe that ‘diversity is good’ conclude that although highly skilled migrants are culturally different from them, the difference is superficial. Differences are perceived as enriching and as contributing to a more sustainable society. Managers also become more open-minded and motivated to work towards inclusion, as well as see integration as a two-way process.

The present author could not agree more with Liu-Farrer’s (2023) remark that highly skilled migrants are being ineffectively used in Japan. Interior frontiers have stood in the way, and addressing and eradicating these boundaries is one way to move forward.

Fichte defined an interior frontier as a boundary between those that belong to the nation and those that do not, and Stoler emphasised that such boundaries result in inequalities ( Stoler, 2017), which is what is happening in the hiring process in Japan. Non-Japanese applicants are less likely to be called back for a second round of interviews after controlling for human capital ( Igarashi and Mugiyama, 2023). Employers’ prejudice against non-Japanese points to boundaries between insiders and outsiders ( Lems, 2020a, 2020b).

Lems (2020a, 2020b) observed in her work that although interior frontiers are less visible than external ones, they have a great deal of power over the lives of individuals, which can be seen in Japanese workplaces when highly skilled migrants are pressurised to assimilate culturally, thought of as imperfect substitutes for Japanese employees, and have their skills and abilities undervalued. These directly impact and have tremendous power over their everyday lives.

Lems also noted that the process of integration for young asylum seekers is one-way, meaning that they have to submit to Swiss rules while the Swiss do not have to do anything or show flexibility in accommodating them. The same can be observed in Japanese workplaces, where highly skilled migrants are required to follow Japanese practices while their employers do little to accommodate them. In the JILPT (2014) publication which cited employers stating Japanese language skills are a precondition for employment, it is clear that they expect one-way submission to Japanese rules on the part of migrants.

Ethnocentric attitudes influenced by *Nihonjinron* manifest themselves too, especially *Nihonjinron*’s emphasis on homogeneity. Japan culture and customs, as well as people, are thought to be unique to the point of being superior to others, which may explain employers’ unwillingness to make exceptions for highly skilled migrants regarding Japanese workplace practices or in wanting them to behave like Japanese employees. Superiority can also result in prejudice against non-Japanese and preference for Japanese employees. The belief that Japan should be homogeneous is pervasive, as can be observed in the forced assimilation of highly skilled migrants in the workplace and the preference for Japanese employees.

The first step to take in moving forward is to challenge the discourse of Japanese homogeneity, which is inaccurate and inappropriate. Indigenous people in Hokkaido and Okinawa have lived in the country for a long time alongside the Japanese, and more recently, the Koreans and Chinese. With globalisation, more migrants are arriving. Japan is clearly not homogeneous. While there is no question that Japanese culture and customs are unique, other cultures and customs are unique too and have their strengths. Japan would benefit far more from being open, flexible and accommodating to highly skilled migrants.

## Data Availability

No data are associated with this article.

## References

[ref1] Becker GS : *The Economics of Discrimination.* Chicago: The University of Chicago Press;1957.

[ref2] Befu H : *Hegemony of Homogeneity.* Melbourne: Trans Pacific Press;2001.

[ref3] Conrad H Meyer-Ohle H : Overcoming the ethnocentric firm? – foreign fresh university graduate employment in Japan as a new international human resource development method. *Int. J. Hum. Resour. Manag.* 2019;30(17):2525–2543. 10.1080/09585192.2017.1330275

[ref4] Cox T : The multicultural organization. *Executive.* 1991;5(2):34–47. 10.5465/ame.1991.4274675

[ref5] Desai-Trilokekar R Thomson K El Masri A : *International Students As Ideal Immigrants: Ontario Employers’ Perspective.* Toronto: York University;2016.

[ref6] Friesen M Ingram S : Advancing intercultural competency: Canadian engineering employers’ experiences with immigrant engineers. *Eur. J. Eng. Educ.* 2013;38:219–227. 10.1080/03043797.2013.766677

[ref7] Ghosh D Gonzalez JA Sekiguchi T : Different feathers embedding together: Integrating diversity and organizational embeddedness. *J. Manag. Stud.* 2023:1–29. 10.1111/joms.12984

[ref8] Haghirian P : Japan’s employment system and human resource management – coping with increasing adjustment pressures. *Contemporary Japan.* 2022;34(1):3–12.

[ref9] Hof H : Intersections of race and skills in European migration to Asia: Between white cultural capital and “passive whiteness”. *Ethn. Racial Stud.* 2021;44(11):2113–2134. 10.1080/01419870.2020.1822535

[ref10] Hof H Tseng Y-F : When “global talents” struggle to become local workers: The new face of skilled migration to corporate Japan. *Asian Pac. Migr. J.* 2021;29(4).

[ref11] Homan AC Hollenbeck JR Humphrey SE : Facing differences with an open mind: Openness to experience, salience of intragroup differences, and performance of diverse work groups. *Acad. Manag. J.* 2008;51(6):1204–1222. 10.5465/amj.2008.35732995

[ref12] Igarashi A Mugiyama R : Whose tastes matter? Discrimination against immigrants in the Japanese labour market. *J. Ethn. Migr. Stud.* 2023;49:3365–3388. 10.1080/1369183X.2022.2163230

[ref13] Joppke C : *Neoliberal Nationalism: Immigration and the Rise of the Populist Right.* Cambridge: Cambridge University Press;2021.

[ref14] Joshi A : The influence of organizational demography on the external networking behavior of teams. *Acad. Manag. Rev.* 2006;31(3):583–595. 10.5465/amr.2006.21318919

[ref15] Lems A : Being inside out: the slippery slope between inclusion and exclusion in a Swiss educational project for unaccompanied refugee youth. *J. Ethn. Migr. Stud.* 2020a;46(2):405–422. 10.1080/1369183X.2019.1584702

[ref16] Lems A : Phenomenology of exclusion: Capturing the everyday thresholds of belonging. *Social Inclusion.* 2020b;8(4):116–125. 10.17645/si.v8i4.3282

[ref17] Liu-Farrer G : The logics of staying for highly skilled Asian migrants in Japan. *Asian Pac. Migr. J.* 2023;32:105–128. 10.1177/01171968231168137

[ref18] Liu-Farrer G Green AE Ozgen C : Immigration and labor shortages: Learning from Japan and the United Kingdom. *Asian Pac. Migr. J.* 2023;32:336–361. 10.1177/01171968231188532

[ref19] Liu-Farrer G Shire K : Who are the fittest? The question of skills in national employment systems in an age of global labour mobility. *J. Ethn. Migr. Stud.* 2021;47(10):2305–2322. 10.1080/1369183X.2020.1731987

[ref20] Morillas M Romani L : Ideology, doxa and critical reflexive learning: The possibilities and limits of thinking that ‘diversity is good’. *Manag. Learn.* 2023;54(4):511–530. 10.1177/13505076221074632

[ref21] Morita L : Why Japan isn’t more attractive to highly-skilled migrants. *Cogent Social Sciences.* 2017;3(1306952):1–12. 10.1080/23311886.2017.1306952

[ref22] Morita L : Japanese norms in Japanese workplaces. *F1000 Res.* 2022;11(1210):1–10.

[ref23] Morita L : Interior frontiers in Japanese blue-collar workplaces. *SSRN.* 2023:1–14. 10.2139/ssrn.4378488

[ref25] Oishi N : Redefining the “highly skilled”: The Points-Based System for highly skilled professionals in Japan. *Asian Pac. Migr. J.* 2014;23(4):421–450. 10.1177/011719681402300406

[ref26] Rear D : A critical analysis of Japanese identity discourses: Alternatives to the hegemony of *Nihonjinron.* *Asian Studies.* 2017;53(2):1–27.

[ref27] Sekiguchi T Froese FJ Iguchi C : International human resource management of Japanese multinational corporations: Challenges and future directions. *Asian Bus. Manag.* 2016;15(2):83–109. 10.1057/abm.2016.5

[ref28] Shachar A : The race for talent: Highly skilled migrants and competitiveimmigration regimes. *N. Y. Univ. Law Rev.* 2006;81(1):148–206.

[ref29] Shore LM Randel AE Chung BG : Inclusion and diversity in work groups: A review and model for future research. *J. Manag.* 2011;37(7):1262–1289. 10.1177/0149206310385943

[ref30] Stoler AL : *“Interior frontiers” as political concept, diagnostic, and dispositif.* Society for Cultural Anthropology Editor’s Forum;2017. Reference Source

[ref31] Stoler AL : Interior frontiers: Diagnostic and dispositive. *Political Concepts: A Critical Lexicon.* 2018. Reference Source

[ref32] The Japan Institute for Labour Policy and Training (JILPT): *Labour Situation in Japan and its Analysis: General Overview 2013/2014.* Tokyo: JILPT;2014.

[ref33] Riemsdijk M van Basford S : Integration of highly skilled migrants in the workplace: A multi-level framework. *J. Int. Migr. Integr.* 2022;23:633–654. 10.1007/s12134-021-00845-x

[ref34] Wakisaka D : *Labyrinth of highly skilled migration in Japan.* University of Bristol;2018. PhD thesis.

