# Supplementary material for: Interior frontiers and highly skilled migrants' work-related challenges in Japan
Source: F1000Res. 2024 Apr 29;13:420. [Version 1] doi: 10.12688/f1000research.147767.1 (PMC11112306; doi:10.12688/f1000research.147767.1)
Supplement: Peer review history

# Reviewer report: Reviewer response for version 1

**DOI:** 10.5256/f1000research.162001.r270679

**Published:** 2024-05-22

**Authors:** Yunchen Tian

**Affiliations:** 1 Department of History and Political Science, Saint Martin's University, Lacey, Washington, USA

Morita argues that even highly skilled workers in Japan, enjoying supposedly privileged visas and migration pathways, face a variety of barriers within Japan. Deeming them internal frontiers, Morita argues that they arise from ethnocentric attitudes, antiquated human resources and managerial practices, and other organizational dynamics in Japanese corporate culture.

 Morita's argument can be simply distilled into identifying the relevance of Stoler's concept of "interior frontiers", itself drawn for the work of Johann Fichte, in conceptualizing the challenges faced by skilled migrants to Japan today.

 This modest argument is suitable for an opinion piece within your journal.

 I do not take issue with any of the argumentation or evidence. However, I suggest that Morita consider the use of the term "frontier" versus another, such as "border". As Stoler admits, the translation from Fichte's original German allows for ambiguity in this regard. She herself uses "interior border" and "interior frontier" interchangeably throughout her chapter on the term.

 However, in contemporary academic literature, "border" and "frontier" have increasingly developed differing nuances. For example, the work of Thomas Nail on the border identifies frontier as having the connotation of (the prospect for) future (often colonial) expansion. (Nail T, 2016 [Ref 1])

 That nuance doesn't seem to exist in Morita's use of the term, so I would suggest that Morita use the term "internal borders" more so than "internal frontiers".

Is the topic of the opinion article discussed accurately in the context of the current literature?

Yes

Are arguments sufficiently supported by evidence from the published literature?

No

Are all factual statements correct and adequately supported by citations?

Yes

Are the conclusions drawn balanced and justified on the basis of the presented arguments?

Yes

Reviewer Expertise:

migration, race and ethnicity, border studies, Japan

I confirm that I have read this submission and believe that I have an appropriate level of expertise to confirm that it is of an acceptable scientific standard.

## References

[1] : Border Kinopower.2016; 10.1093/acprof:oso/9780190618643.003.0002 21-44 10.1093/acprof:oso/9780190618643.003.0002

# Reviewer report: Reviewer response for version 1

**DOI:** 10.5256/f1000research.162001.r270674

**Published:** 2024-05-13

**Authors:** Stephen R Nagy

**Affiliations:** 1 Department of Politics and International studies, International Christian University, Tokyo, Japan

The author uses an interesting framework, interior frontier to discuss the challenges of highly skilled migrants. 

 It covers the essential literature such as Liu-Farrer, Oishi and others.

 It is balanced and not normative, sticking to the data and academic literature. 

 The only squabble that I have is that the problems faced by highly skilled overlap heavily with the non-skilled/ low skilled migrants. 

 The exclusive nature of Japan's ethno-centric identity based on insider and outsider groups at all levels in society creates conditions where everyone including Japanese citizens are negotiating the interior frontier when they engage with groups that are not their insider group.

Is the topic of the opinion article discussed accurately in the context of the current literature?

Yes

Are arguments sufficiently supported by evidence from the published literature?

Yes

Are all factual statements correct and adequately supported by citations?

Yes

Are the conclusions drawn balanced and justified on the basis of the presented arguments?

Yes

Reviewer Expertise:

I conducted research on Japan's migration policies in the past

I confirm that I have read this submission and believe that I have an appropriate level of expertise to confirm that it is of an acceptable scientific standard.
